
JOURNAL INFORMATION
==============================
NLM Title Abbreviation: Innov Aging
Journal ID: Innov Aging
NLM Journal ID: 101703706
Journal ID: innovateage

Innovation in Aging

EISSN: 2399-5300
Publisher: Oxford University Press

ARTICLE INFORMATION
==============================
PMCID: PMC11690415
DOI: 10.1093/geroni/igae098.1141
Article ID: igae098.1141
Article version: 1
Subjects: Abstracts, Session 3020 (Biological Sciences Invited Symposium), AcademicSubjects/SOC02600

ADVANCES IN CELLULAR SENESCENCE

Passos Joao Chair

Publication date: 2024 Dec
Electronic publication date: 2024 Dec 31
Volume: 8
Issue: Suppl 1
Issue title: Program Abstracts from the GSA 2024 Annual Scientific Meeting, “The Fortitude Factor”
First page: 350
Last page: 350
Copyright: © The Author(s) 2024. Published by Oxford University Press on behalf of The Gerontological Society of America.
Copyright year: 2024
License: This is an Open Access article distributed under the terms of the Creative Commons Attribution License (https://creativecommons.org/licenses/by/4.0/), which permits unrestricted reuse, distribution, and reproduction in any medium, provided the original work is properly cited.
License URL: https://creativecommons.org/licenses/by/4.0/

Page count: 1

==============================
Abstract

